# Dutch normative data and psychometric properties for the Distress Thermometer for Parents

**DOI:** 10.1007/s11136-016-1405-4

**Published:** 2016-09-02

**Authors:** Hedy A. van Oers, Sasja A. Schepers, Martha A. Grootenhuis, Lotte Haverman

**Affiliations:** 1 Psychosocial Department G8-136, Emma Children’s Hospital/Academic Medical Center, PO Box 22660, 1100 DD Amsterdam, The Netherlands; 2 Princess Máxima Center for Pediatric Oncology, Utrecht, The Netherlands

**Keywords:** Parents, Pediatrics, Screening, Psychosocial functioning, Distress, Questionnaire

## Abstract

**Purpose:**

The aim of this study was to provide Dutch normative data for the Distress Thermometer for Parents (DT-P) and to assess internal consistency and known-groups validity.

**Methods:**

A sample of 1421 parents (60.7 % mothers), representative of the Dutch population, completed online sociodemographic questionnaire and the DT-P, which includes a thermometer (0 (no distress) to 10 (extreme distress), ≥4 clinically elevated distress) and everyday problems across six problem domains (practical, social, emotional, physical, cognitive, and parenting). Internal consistency was calculated using Cronbach’s alphas. Known-groups validity was assessed by comparing parents of a child with a chronic condition (*N* = 287, 20.2 %) with parents of healthy children, using Mann–Whitney *U* tests and Chi-square tests.

**Results:**

The DT-P showed acceptable internal consistency (Cronbach’s alphas = .52–.89). Parents of a child with a chronic condition more often reported clinically elevated distress than parents of healthy children (53.0 versus 38.2 %, *p* < .001). Also, on all domains they reported more problems (*p* = .000–.022). Normative scores for mothers and fathers separately were provided.

**Conclusion:**

The DT-P distinguishes well between parents of a child with and without a chronic condition. With the current norms available, distress can be evaluated in parents of a child with a chronic condition compared to parents of healthy children in pediatric clinical practice.

**Electronic supplementary material:**

The online version of this article (doi:10.1007/s11136-016-1405-4) contains supplementary material, which is available to authorized users.

## Introduction

In the Netherlands, approximately 14 % of children are growing up with a chronic health condition [1]. A similar percentage was found in the USA; chronic conditions of any type affect 15–18 % of children and adolescents [2]. Such a condition can affect the whole family. Over the past decade, an increasing number of studies have reported on parental functioning in pediatrics [3]. Parents of a chronically ill child are at risk of a lower health-related quality of life (HRQoL), experience more posttraumatic stress symptoms (PTSS), and report higher levels of distress than parents of healthy children. In addition, previous studies show that parental psychosocial problems influence the well-being of the child. For example, maternal depression negatively influences the child’s adherence which has an impact on the disease severity [4–6]. Therefore, it is important to pay attention to the well-being of these parents, in order to provide appropriate psychosocial interventions.

To efficiently screen whether parents of a child with a chronic condition need and want support, the Distress Thermometer for Parents (DT-P) was developed [7] and found to be an internally consistent and empirically supported instrument (construct validity) for identifying parental distress in parents of children with a chronic condition. However, within the general population, the reliability (internal consistency), known-groups validity (the extent to which a measurement is sensitive to differences in various groups) and normative data are lacking. Therefore, the aims of this study were to collect Dutch normative data, and to assess internal consistency and known-groups validity.

## Methods

### Participants and procedures

Data collection of the DT-P was part of a larger study with the objective of establishing normative data for several questionnaires, completed by parents, used in pediatrics. In November and December 2014, parents (one respondent per family) representative of the general Dutch population were invited by e-mail to participate. Online data collection was carried out by Dutch market research agency ‘Taylor Nelson Sofres Netherlands Institute for Public Opinion’ (TNS NIPO). The sample was stratified from their database based on Dutch population figures regarding key demographics. With the objective of obtaining around 1400 respondents (response rate of 60 %), a stratified sample of 2299 parents was drawn from the database. Prior to completing the questionnaires, informed consent was obtained from all participants. The study was performed with permission of and in accordance with the regulations of the Medical Ethics Committee of the Academic Medical Center, Amsterdam, the Netherlands.

### Measures

#### Sociodemographics

Information regarding parental age, gender, educational level, country of birth, and employment was provided by TNS NIPO for participants as well as non-participants. Participants completed a study-specific sociodemographic questionnaire about their marital status and number of children. The chronic condition of the children was reported by parents with the question: *‘Does your child have a chronic health condition? If Yes, please specify’* and later categorized by a pediatrician in the Emma Children’s Hospital. To decide whether a child had a chronic condition, the criteria of Mokkink et al. were used [1], which is: (1) it occurs in children aged 0 up to 18 years; (2) the diagnosis is based on medical scientific knowledge and can be established using reproducible and valid methods or instruments according to professional standards; (3) it is not (yet) curable or, for mental health conditions, if it is highly resistant to treatment; and (4) it has been present for longer than 3 months or it will, very probably, last longer than 3 months, or it has occurred three times or more during the past year and will probably reoccur.

#### DT-P

The DT-P is a well-validated, brief screening instrument that is used in clinical practice in the Netherlands to identify distress and everyday problems in parents of children with a chronic condition [7]. In pediatric oncology a DT-P is developed in the USA; however, this instrument has not been studied in a large sample of parents of children with several chronic health conditions. Therefore Kazak et al. [8] and Patel et al. [9] emphasized the necessity of developing a DT especially for parents of a chronically ill child and to examine its diagnostic utility in a large sample. The Dutch DT-P is an adaptation of the Dutch version of the Distress Thermometer, a screening tool in standard adult oncology practice [10, 11]. The adaptation of the Distress Thermometer for the use in daily clinical practice consisted mainly of deleting physical items—because parents are not patients—and of adding items on parenting problems (interacting with the child, independence of the child, following advice about treatment of the child) [7]. The DT-P consists of (1) a ‘thermometer’ ranging from 0 (no distress) to 10 (extreme distress) on which parents rate their overall distress in the past week, where a thermometer score of 4 or higher indicates clinically elevated distress, (2) a problem list which inquires the occurrence of 37 (child age < 2 years) or 34 (child age ≥ 2 years) everyday problems over the past week across six problem domains (practical, social, emotional, physical, cognitive, and parenting), where problem domain scores are the sum of item scores (yes = 1, no = 0) within that problem domain, and (3) additional questions concerning: perceived support from surroundings, perceived lack of understanding from people concerning their situation, parental chronic illness, and whether or not the parent would like to talk to a professional about his or her situation. In clinical practice, the DT-P is used in daily clinical practice to screen for parental distress and to refer to psychosocial care, but also as part of standard battery in clinics [12].

### Statistical analyses

The Statistical Package for Social Sciences (SPSS) version 23 was used for all statistical analyses. First, differences in sociodemographic data (age, gender, country of birth, education level, and employment status) between participants and non-participants were compared, with the information provided by TNS NIPO, using a *t* test and Chi-square tests.

Second, to determine internal consistency of the DT-P problem domains, Cronbach’s alpha coefficients were calculated based on the average inter-item correlation [13]. Because of the diversity of constructs being measured in psychology, Cronbach’s alphas with values below .70 can realistically be expected. Therefore, subscales with Cronbach’s alpha of ≥.60 were considered acceptable [14].

Third, known-groups validity was determined by testing differences (in all parents, and mothers and fathers separately) in DT-P scores between parents of children with a chronic condition and parents of children without a chronic condition using *t* tests (for mean thermometer score), Mann–Whitney *U* tests (for problem domain scores) and Chi-square tests (for clinical distress and everyday problems).

## Results

In total, 1421 parents (response rate 61.8 %) participated, including 287 (20.2 %) parents of one or more children with a chronic condition. The 10 most reported chronic conditions of children (all chronically ill children) were autism/PDD-NOS (17.1 %), asthma/lung problems (15.3 %), ADHD (10.8 %), eczema/skin conditions (10.1 %), allergies (8.4 %), intellectual disability (4.9 %), skeletal or bone abnormality/cleft (4.9 %), muscle disorder (3.5 %), gastrointestinal disease (3.5 %), and heart disease (2.8 %). The sociodemographics did not differ between the participants (*N* = 1421) and the non-participants within the total stratified sample (*N* = 2299), except for gender [relatively more fathers among the non-participants (43.8 %) compared to participants (39.3 %), *p* = .035].

Cronbach’s alpha coefficients of the DT-P problem domains were: for the practical problem domain .60, social problem domain .52, emotional problem domain .79, physical problem domain .68, cognitive problem domain .69, parenting problem domain <2 years .63, parenting problem domain ≥2 years .70. Cronbach’s alpha for the total problem score (5 domains) was .88, total problem score including parenting problem domain <2 years (6 domains) .87, total problem score including parenting problem domain ≥2 years (6 domains) .89.

Table 1 shows the sociodemographics of the participants and non-participants. Table 2 contains the DT-P scores of all participants, subdivided by parents of children with and without a chronic condition. Parents of a child with a chronic condition reported more often than parents of healthy children clinically elevated distress (53.0 versus 38.2 %, *p* < .001) and a higher mean thermometer score (4.2 versus 3.4, *p* < .001). Also, on all problem domain scores they reported more problems (*p* < .0001–.022), except for the parenting problem domain for parents of children <2 years (*p* = .544), and they significantly differed on 23 of the 34 everyday problems when their child was ≥2 years or 18 of the 37 everyday problems when their child <2 years. In addition, parents of a child with a chronic condition less often indicated that they received enough social support from surroundings (*p* < .001), more often indicated that people around them reacted with a lack of understanding (*p* < .001), and that they would like to talk to a professional about their situation (*p* = .004).

**Table 1:** Sociodemographic characteristics of participant and non-participants

	Participants (*N* = 1421)	Non-participants (*N* = 878)	*p*
Child			
Age in years, *M* (SD), range	8.07 (5.59)	8.06 (5.13), .2–19.0	.936
Female gender (%)	46.9	48.9	.340
Parents*			
Age in years, *M* (SD), range	40.5 (7.1), 18.1–75.3	40.20 (6.87), 24.3–66.2	.287
Female gender (%)	60.7	56.2	.035
Born in the Netherlands^a^ (%)	96.4	95.8	.259
Educational level^b^ (%)			
Low	16.3	19.9	.060
Intermediate	42.9	44.1	
High	40.0	35.4	
Paid employment^c^ (%)	83.8	82.2	.267
Marital status^d,e^ (%)			
Married/living together	91.8	–	–
Single/separated/widow	8.0	–	–
Number of children^e^ (%)			
1	17.7	–	–
2	56.5	–	–
≥3	25.8	–	–

* 19 parents indicated to be a foster or a step parent

^a^ *N* = 1 parent (.1 %) answered ‘do not know/do not want to tell’

^b^Highest educational level completed. Low: primary education, lower vocational education, lower or middle general secondary education; Intermediate: middle vocational education, higher secondary education, pre-university education; High: higher vocational education, university. *N* = 11 participating parents (.8 %) and *N* = 5 non-participating parents (.6 %) answered ‘do not know/do not want to tell’

^c^ *N* = 4 participating parents (.3 %) and *N* = 6 non-participating parents (.7 %) answered ‘do not know/do not want to tell’

^d^ *N* = 2 parents (.1 %) answered ‘do not know/do not want to tell’

^e^Data not applicable for non-participants

**Table 2:** Distress Thermometer score, problem domain scores, and item scores of all participants, and subdivided in parents of children with (CC) and without (No CC) chronic conditions

	All participants *N* = 1421	CC *N* = 287	No CC *N* = 1134	*p*
Thermometer score				
Clinical (%)	41.2	53.0	38.2	<.**0001**
Mean (SD)	3.4 (2.7)	4.2 (2.9)	3.2 (2.7)	<.**0001**
Median (range)	3 (0–10)	4 (0–10)	2 (0–10)	<.**0001**
Total problem scores, medians (range)				
Total of 5 problem domains	4 (0–28)	5 (0–26)	3 (0–28)	<.**0001**
Total with <2 years parenting	5^a^ (0–27)	9^c^ (1–21)	5^e^ (0–27)	.**028**
Total with ≥2 years parenting	4^b^ (0–33)	5^d^ (0–28)	3^f^ (0–33)	<.**0001**
Practical problems, median (range)	1 (0–7)	1 (0–6)	0 (0–7)	.**001**
Housing (%)	5.0	5.9	4.8	.420
Work/study (%)	24.8	21.6	25.6	.164
Finances/insurance (%)	16.5	19.5	15.8	.129
Housekeeping (%)	20.1	29.3	17.7	<.**0001**
Transport (%)	5.1	8.0	4.3	.**011**
Child care/child supervision (%)	9.7	15.7	8.2	<.**0001**
Leisure activities/relaxing (%)	21.3	28.9	19.3	<.**0001**
Social problems, median (range)	0 (0–4)	0 (0–4)	0 (0–4)	<.**0001**
Dealing with (ex)partner (%)	13.3	18.1	12.1	.**007**
Dealing with family (%)	9.6	11.1	9.2	.309
Dealing with friends (%)	3.0	3.8	2.8	.372
Interacting with your child(ren) (%)	12.3	20.9	10.1	<.**0001**
Emotional problems, median (range)	1 (0–9)	1 (0–9)	1 (0–9)	.**001**
Controlling emotions (%)	22.2	26.8	21.1	.**036**
Self-confidence (%)	19.8	24.4	18.6	.**028**
Fears (%)	9.4	11.1	9.0	.264
Depression (%)	29.8	37.3	28.0	.**002**
Feeling tense or nervous (%)	33.7	40.1	32.1	.**011**
Loneliness (%)	7.3	12.2	6.1	<.**0001**
Feelings of guilt (%)	13.8	15.7	13.3	.300
Use of substances (e.g., alcohol, drugs and/or medication) (%)	3.0	3.5	2.8	.554
Intrusive/recurrent thoughts about a specific event (%)	19.1	24.4	17.7	.**010**
Physical problems, median (range)	1 (0–7)	2 (0–7)	1 (0–7)	<.**0001**
Eating (%)	10.1	13.2	9.3	.**045**
Weight (%)	23.8	29.6	22.3	.**009**
Sleep (%)	26.9	29.3	26.3	.307
Fatigue (%)	52.1	56.8	51.0	.078
Out of shape/condition (%)	22.4	31.7	20.1	<.**0001**
Pain (%)	23.9	32.4	21.8	<.**0001**
Sexuality (%)	10.3	11.8	9.9	.326
Cognitive problems, median (range)	0 (0–2)	0 (0–2)	0 (0–2)	<.**0001**
Concentration (%)	17.2	25.1	15.2	<.**0001**
Memory (%)	21.4	31.7	18.8	<.**0001**
Parenting problems <2 years, median (range)	0^a^ (0–6)	0^c^ (0–4)	0^e^ (0–6)	.544
Feeling connected with your child (%)	1.9	3.7	1.6	.451
Caring for your child (%)	2.8	3.7	2.7	.762
Feeding your child (%)	12.6	7.4	13.4	.383
Development of your child (%)	6.5	11.1	5.9	.304
Following advice about treatment/giving medication (%)	2.3	3.7	2.1	.615
Your child’s sleeping (%)	23.4	25.9	23.0	.737
Behavior/crying of your child (%)	17.3	18.5	17.1	.857
Parenting problems ≥2 years, median (range)	0^b^ (0–5)	0^d^ (0–5)	0^f^ (0–5)	<.**0001**
Dealing with your child (%)	12.0	17.4	10.5	.**002**
Dealing with the feelings of your child (%)	11.6	20.2	9.2	<.**0001**
Talking about the disease/consequences with your child* (%)	4.1	8.1	2.9	<**.0001**
Independence of your child (%)	9.1	14.3	7.6	.**001**
Following advice about treatment/giving medication (%)	4.8	10.5	3.3	<.**0001**
Additional questions				
Enough support from surroundings (%)	90.2	80.8	92.6	<.**0001**
People react with a lack of understanding (%)	13.4	23.7	10.8	<.**0001**
Do you have a (chronic) illness yourself (%)	21.4	35.9	17.7	<.**0001**
Would like to talk to a professional about situation—yes/maybe (%)	16.7	22.3	15.3	.**004**

Mean thermometer score was analyzed with *t* test. Median thermometer score, total problem scores, and problem domain scores were analyzed with Mann–Whitney *U* tests. The presence of a clinical thermometer score and of reported problems (individual items) were analyzed with Chi-square tests. Significant differences at *p* < .05 are presented in bold

* Parents could also indicate that ‘talking about the disease/consequences with your child’ was not applicable. This was rated as 0: not a problem

^a^ *N* = 214 (19 parents did not complete this domain)

^b^ *N* = 1176 (12 parents did not complete this domain)

^c^ *N* = 27 (2 parents did not complete this domain)

^d^ *N* = 258

^e^ *N* = 187 (17 parents did not complete this domain)

^f^ *N* = 918 (12 parents did not complete this domain)

Normative scores for mothers and fathers separately were provided; both subdivided by parents of children with and without a chronic condition (see Supplemental Tables 3 and 4). These results showed similar findings to the total group of participants.

## Discussion

This study aimed to provide Dutch normative data for the Distress Thermometer for Parents (DT-P) and to assess internal consistency and known-groups validity. Acceptable reliability was found for the DT-P problem domains and the DT-P differentiated between parents of a child with a chronic condition and parents of healthy children, except for the parenting problem domain for parents of children <2 years. This might be explained by a smaller sample size. In addition, when children are very young, parents might experience similar problems, regarding feeding and sleeping, for example, compared to parents of healthy children.

In the initial study undertaken to develop the DT-P, 47.3 % in a sample of parents of a child with a chronic condition reported an elevated distress score [7], which is significantly lower compared to the findings in this study (53.0 %). Possible explanations could be that in the initial study many children (20.9 %) were in a follow-up trajectory after admission to the NICU/PICU or after a history of Kawasaki disease, and therefore differed from this group of children with a chronic condition. Furthermore, that population did not include psychiatric chronic conditions, which were included in this normative study.

A limitation of this study is that chronic condition of the child was based on parent report rather than on pediatrician report. Therefore, it is possible that our sample contained a slightly different type of chronic condition than encountered in the general Dutch population. Furthermore, the Cronbach’s alpha of the social problem domain was rather weak (.52) and this might have to with the fact that the items in this subscale (dealing with (ex)partner, family, friends, and interacting with your child(ren) do not necessarily have to be related to each other [14]). Cautiousness is warranted while interpreting the score on this subscale.

In conclusion, with the current normative data available, distress can be evaluated in parents of a child with a chronic condition compared to parents of healthy children in pediatric clinical practice.

## Electronic supplementary material

Below is the link to the electronic supplementary material.

Supplementary material 1 (DOC 102 kb)

Supplementary material 2 (DOC 119 kb)
