# Supplementary material for: Dutch normative data and psychometric properties for the Distress Thermometer for Parents
Source: Qual Life Res. 2016 Sep 2;26(1):177–82. doi: 10.1007/s11136-016-1405-4 (PMC5243897; doi:10.1007/s11136-016-1405-4)
Supplement: Supplementary file 1 — Supplementary material 1 (DOC 102 kb) [file 11136_2016_1405_MOESM1_ESM.doc]

Table 3 - Supplemental

*Distress Thermometer score, problem domain scores and item scores of all mothers, and subdivided in mothers of children with (CC) and without (No CC) chronic conditions.*

|  | **All mothers**  **N=862** | **CC**  **N=191** | **No CC**  **N=671** | ***p*** |  |
| --- | --- | --- | --- | --- | --- |
| Thermometer score |  |  |  |  |  |
| Clinical, % | 45.2 | 55.5 | 42.3 | .**001** |  |
| Mean (SD) | 3.7 (2.8) | 4.4 (2.9) | 3.5 (2.7) | **<.0001** |  |
| Median (range) | 3 (0-10) | 4 (0-10) | 3 (0-10) | **<.0001** |  |
| Total problem scores, medians (range) |  |  |  |  |  |
| Total of 5 problem domains | 5 (0-26) | 6 (0-26) | 4 (0-25) | .**001** |  |
| Total with <2 years parenting | 7^a^ (0-27) | 9^c^ (1-15) | 6^e^ (0-27) | .401 |  |
| Total with ≥2 years parenting | 5^b^ (0-29) | 6^d^ (0-28) | 4^f^ (0-29) | **<.0001** |  |
| Practical problems, median (range) | 1 (0-7) | 1 (0-6) | 1 (0-7) | .**008** |  |
| Housing, % | 5.8 | 6.8 | 5.5 | .500 |  |
| Work/study, % | 24.6 | 22.0 | 25.3 | .343 |  |
| Finances/insurance, % | 17.3 | 19.4 | 16.7 | .387 |  |
| Housekeeping, % | 25.1 | 37.2 | 21.6 | **<.0001** |  |
| Transport, % | 5.5 | 8.4 | 4.6 | **.044** |  |
| Child care/child supervision, % | 11.6 | 16.8 | 10.1 | **.012** |  |
| Leisure activities/relaxing, % | 24.4 | 31.4 | 22.4 | **.010** |  |
| Social problems, median (range) | 0 (0-4) | 0 (0-4) | 0 (0-4) | .**041** |  |
| Dealing with (ex)partner, % | 13.8 | 18.8 | 12.4 | .**022** |  |
| Dealing with family, % | 11.3 | 12.6 | 10.9 | .515 |  |
| Dealing with friends, % | 3.9 | 4.7 | 3.7 | .537 |  |
| Interacting with your child(ren), % | 13.5 | 19.4 | 11.8 | .**007** |  |
| Emotional problems, median (range) | 1 (0-9) | 1 (0-9) | 1 (0-9) | .094 |  |
| Controlling emotions, % | 27.0 | 25.7 | 27.4 | .628 |  |
| Self-confidence, % | 23.4 | 26.2 | 22.7 | .310 |  |
| Fears, % | 11.0 | 12.0 | 10.7 | .610 |  |
| Depression, % | 33.4 | 38.7 | 31.9 | .077 |  |
| Feeling tense or nervous, % | 37.0 | 40.3 | 36.1 | .283 |  |
| Loneliness, % | 9.0 | 13.6 | 7.7 | .**001** |  |
| Feelings of guilt, % | 17.6 | 18.3 | 17.4 | .776 |  |
| Use of substances (e.g. alcohol, drugs and/or medication) , % | 3.0 | 4.2 | 2.7 | .283 |  |
| Intrusive/recurrent thoughts about a specific event, % | 21.8 | 26.7 | 20.4 | .064 |  |
| Physical problems, median (range) | 2 (0-7) | 2 (0-7) | 2 (0-7) | .**031** |  |
| Eating, % | 13.1 | 15.7 | 12.4 | .228 |  |
| Weight, % | 28.2 | 35.1 | 26.2 | .**016** |  |
| Sleep, % | 30.2 | 31.9 | 29.7 | .545 |  |
| Fatigue, % | 57.2 | 62.3 | 55.7 | .106 |  |
| Out of shape/condition, % | 24.5 | 37.2 | 20.9 | **<.0001** |  |
| Pain, % | 27.4 | 38.2 | 24.3 | **<.0001** |  |
| Sexuality, % | 10.7 | 11.0 | 10.6 | .870 |  |
| Cognitive problems, median (range) | 0 (0-2) | 0 (0-2) | 0 (0-2) | **<.0001** |  |
| Concentration, % | 20.0 | 27.2 | 17.9 | .**004** |  |
| Memory, % | 25.6 | 37.2 | 22.4 | **<.0001** |  |
| Parenting problems <2 years, median (range) | 0^a^ (0-6) | 0^c^ (0-4) | 0^e^ (0-6) | .686 |  |
| Feeling connected with your child, % | 2.5 | 5.6 | 1.9 | .363 |  |
| Caring for your child, % | 2.5 | 5.6 | 1.9 | .363 |  |
| Feeding your child, % | 14.9 | 5.6 | 16.5 | .228 |  |
| Development of your child, % | 7.4 | 11.1 | 6.8 | .520 |  |
| Following advice about treatment/giving medication, % | 3.3 | 5.6 | 2.9 | .563 |  |
| Your child’s sleeping, % | 25.6 | 33.3 | 24.3 | .416 |  |
| Behavior/crying of your child, % | 16.5 | 16.7 | 16.5 | .986 |  |
| Parenting problems ≥2 years, median (range) | 0^b^ (0-5) | 0^d^ (0-5) | 0^f^ (0-5) | **<.0001** |  |
| Dealing with your child, % | 12.4 | 17.3 | 10.9 | .**025** |  |
| Dealing with the feelings of your child, % | 12.4 | 22.0 | 9.5 | **<.0001** |  |
| Talking about the disease/consequences with your child, % ^c^ | 4.6 | 9.2 | 3.1 | .**001** |  |
| Independence of your child, % | 9.4 | 15.0 | 7.6 | .**004** |  |
| Following advice about treatment/giving medication, % | 5.0 | 9.8 | 3.5 | .**001** |  |
| Additional questions |  |  |  |  |  |
| Enough support from surroundings, % | 89.2 | 79.1 | 92.1 | **<.0001** |  |
| People react with a lack of understanding, % | 14.3 | 24.6 | 11.3 | **<.0001** |  |
| Do you have a (chronic) illness yourself, % | 24.0 | 37.2 | 20.3 | **<.0001** |  |
| Would like to talk to a professional about situation - Yes/Maybe, % | 18.0 | 20.9 | 17.1 | .227 |  |

*Notes.* Mean thermometer score was analyzed with t-test. Median thermometer score, total problem scores and problem domain scores were analyzed with Mann-Whitney U-tests. The presence of a clinical thermometer score and of reported problems (individual items) were analyzed with Chi^2^ tests. Signiﬁcant differences at *p* < .05 are presented in bold.

^*^ Parents could also indicate that ‘talking about the disease/consequences with your child’ was not applicable. This was rated as 0: not a problem.

^a^ N=121 (8 mothers did not complete this domain), ^b^ N= 723 (10 mothers did not complete this domain), ^c^ N=18, ^d^ N=173, ^e^ N=103 (8 mothers did not complete this domain), ^f^ N=550 (10 mothers did not complete this domain)
