# Supplementary material for: Dutch normative data and psychometric properties for the Distress Thermometer for Parents
Source: Qual Life Res. 2016 Sep 2;26(1):177–82. doi: 10.1007/s11136-016-1405-4 (PMC5243897; doi:10.1007/s11136-016-1405-4)
Supplement: Supplementary file 2 — Supplementary material 2 (DOC 119 kb) [file 11136_2016_1405_MOESM2_ESM.doc]

Table 4 - Supplemental

*Distress Thermometer score, problem domain scores and item scores of all fathers, and subdivided in fathers of children with (CC) and without (No CC) chronic conditions.*

|  | **All fathers**  **N=559** | **CC**  **N=96** | **No CC**  **N=463** | ***p*** |  |
| --- | --- | --- | --- | --- | --- |
| Thermometer score |  |  |  |  |  |
| Clinical, % | 34.9 | 47.9 | 32.2 | .**003** |  |
| Mean (SD) | 3.0 (2.6) | 3.8 (3.0) | 2.8 (2.5) | .**003** |  |
| Median (range) | 2 (0-10) | 3 (0-9) | 2 (0-10) | .**006** |  |
| Total problem scores, medians (range) |  |  |  |  |  |
| Total of 5 problem domains | 2 (0-28) | 4 (0-20) | 2 (0-28) | .**021** |  |
| Total with <2 years parenting | 3^a^ (0-23) | 10^c^ (1-21) | 3^e^ (0-23) | .**043** |  |
| Total with ≥2 years parenting | 2^b^ (0-33) | 4^d^ (0-20) | 2^f^ (0-33) | .054 |  |
| Practical problems, median (range) | 0 (0-7) | 1 (0-6) | 0 (0-7) | .062 |  |
| Housing, % | 3.8 | 4.2 | 3.7 | .816 |  |
| Work/study, % | 25.0 | 20.8 | 25.9 | .295 |  |
| Finances/insurance, % | 15.4 | 19.8 | 14.5 | .189 |  |
| Housekeeping, % | 12.3 | 13.5 | 12.1 | .695 |  |
| Transport, % | 4.5 | 7.3 | 3.9 | .142 |  |
| Child care/child supervision, % | 6.8 | 13.5 | 5.4 | .**004** |  |
| Leisure activities/relaxing, % | 16.5 | 24.0 | 14.9 | .**029** |  |
| Social problems, median (range) | 0 (0-4) | 0 (0-3) | 0 (0-4) | .**001** |  |
| Dealing with (ex)partner, % | 12.5 | 16.7 | 11.7 | .178 |  |
| Dealing with family, % | 7.0 | 8.3 | 6.7 | .566 |  |
| Dealing with friends, % | 1.6 | 2.1 | 1.5 | .686 |  |
| Interacting with your child(ren), % | 10.6 | 24.0 | 7.8 | **<.0001** |  |
| Emotional problems, median (range) | 0 (0-9) | 1 (0-8) | 0 (0-9) | .**002** |  |
| Controlling emotions, % | 14.8 | 29.2 | 11.9 | **<.0001** |  |
| Self-confidence, % | 14.1 | 20.8 | 12.7 | .**038** |  |
| Fears, % | 7.0 | 9.4 | 6.5 | .311 |  |
| Depression, % | 24.3 | 34.4 | 22.2 | .**012** |  |
| Feeling tense or nervous, % | 28.6 | 39.6 | 26.3 | .**009** |  |
| Loneliness, % | 4.7 | 9.4 | 3.7 | .**016** |  |
| Feelings of guilt, % | 7.9 | 10.4 | 7.3 | .309 |  |
| Use of substances (e.g. alcohol, drugs and/or medication) , % | 2.9 | 2.1 | 3.0 | .615 |  |
| Intrusive/recurrent thoughts about a specific event, % | 14.8 | 19.8 | 13.8 | .134 |  |
| Physical problems, median (range) | 1 (0-7) | 1 (0-6) | 1 (0-7) | .383 |  |
| Eating, % | 5.4 | 8.3 | 4.8 | .156 |  |
| Weight, % | 17.0 | 18.8 | 16.6 | .615 |  |
| Sleep, % | 21.8 | 24.0 | 21.4 | .578 |  |
| Fatigue, % | 44.4 | 45.8 | 44.1 | .750 |  |
| Out of shape/condition, % | 19.3 | 20.8 | 19.0 | .680 |  |
| Pain, % | 18.6 | 20.8 | 18.1 | .538 |  |
| Sexuality, % | 9.7 | 13.5 | 8.9 | .157 |  |
| Cognitive problems, median (range) | 0 (0-2) | 0 (0-2) | 0 (0-2) | .**028** |  |
| Concentration, % | 12.9 | 20.8 | 11.2 | .**011** |  |
| Memory, % | 14.8 | 20.8 | 13.6 | .070 |  |
| Parenting problems <2 years, median (range) | 0^a^ (0-5) | 0^c^ (0-2) | 0^e^ (0-5) | .691 |  |
| Feeling connected with your child, % | 1.1 | 0.0 | 1.2 | .742 |  |
| Caring for your child, % | 3.2 | 0.0 | 3.6 | .564 |  |
| Feeding your child, % | 9.7 | 11.1 | 9.5 | .878 |  |
| Development of your child, % | 5.4 | 11.1 | 4.8 | .422 |  |
| Following advice about treatment/giving medication, % | 1.1 | 0.0 | 1.2 | .742 |  |
| Your child’s sleeping, % | 20.4 | 11.1 | 21.4 | .466 |  |
| Behavior/crying of your child, % | 18.3 | 22.2 | 17.9 | .747 |  |
| Parenting problems ≥2 years, median (range) | 0^b^ (0-5) | 0^d^ (0-5) | 0^f^ (0-5) | .**019** |  |
| Dealing with your child, % | 11.3 | 17.6 | 9.8 | .**039** |  |
| Dealing with the feelings of your child, % | 10.2 | 16.5 | 8.7 | .**032** |  |
| Talking about the disease/consequences with your child, % ^*^ | 3.3 | 5.9 | 2.7 | .142 |  |
| Independence of your child, % | 8.6 | 12.9 | 7.6 | .114 |  |
| Following advice about treatment/giving medication, % | 4.6 | 11.8 | 3.0 | .**001** |  |
| Additional questions |  |  |  |  |  |
| Enough support from surroundings, % | 91.8 | 84.4 | 93.3 | .**004** |  |
| People react with a lack of understanding, % | 12.2 | 21.9 | 10.2 | .**001** |  |
| Do you have a (chronic) illness yourself, % | 17.4 | 33.3 | 14.0 | **<.0001** |  |
| Would like to talk to a professional about situation - Yes/Maybe, % | 14.7 | 25.0 | 12.5 | .**002** |  |

*Notes.* Mean thermometer score was analyzed with t-test. Median thermometer score, total problem scores and problem domain scores were analyzed with Mann-Whitney U-tests. The presence of a clinical thermometer score and of reported problems (individual items) were analyzed with Chi^2^ tests. Signiﬁcant differences at *p* < .05 are presented in bold.

^*^ Parents could also indicate that ‘talking about the disease/consequences with your child’ was not applicable. This was rated as 0: not a problem.

^a^ N=93 (11 fathers did not complete this domain), ^b^ N=453 (2 fathers did not complete this domain), ^c^ N=9 (2 fathers did not complete this domain),  ^d^ N=85, ^e^ N=84 (9 fathers did not complete this domain), ^f^ N=368 (2 fathers did not complete this domain)
